# Probe Into the Influence of Crosslinking on CO_2_ Permeation of Membranes

**DOI:** 10.1038/srep40082

**Published:** 2017-01-04

**Authors:** Jinghui Li, Zhuo Chen, Ahmad Umar, Yang Liu, Ying Shang, Xiaokai Zhang, Yao Wang

**Affiliations:** 1 School of Chemistry and Environment, Key Laboratory of Bio-Inspired Smart Interfacial Science and Technology of Ministry of Education, Beihang University, Beijing 100191, PR China; 2 Department of Chemistry, Faculty of Science and Arts and Promising Centre for Sensors and Electronic Devices (PCSED), Najran University, Najran 11001, Kingdom of Saudi Arabia; 3 Beijing Key Laboratory of Radiation Advanced Materials, Beijing Research Center for Radiation Application, Beijing 100015, China

## Abstract

Crosslinking is an effective way to fabricate high-selective CO_2_ separation membranes because of its unique crosslinking framework. Thus, it is essentially significant to study the influence of crosslinking degree on the permeation selectivities of CO_2_. Herein, we report a successful and facile synthesis of a series of polyethylene oxide (PEO)-based diblock copolymers (BCP) incorporated with an unique UV-crosslinkable chalcone unit using Reversible Addition-Fragmentation Chain Transfer Polymerization (RAFT) process. The membranes of as-prepared BCPs show superior carbon dioxide (CO_2_) separation properties as compared to nitrogen (N_2_) after UV-crosslinking. Importantly, the influence of different proportions of crosslinked chalcone on CO_2_ selectivities was systematically investigated, which revealed that CO_2_ selectivities increased obviously with the enhancement of chalcone fractions within a certain limit. Further, the CO_2_ selectivities of block copolymer with the best block proportion was studied by varying the crosslinking time which confirmed that the high crosslinking degree exhibited a better CO_2_/N_2_ (α_CO2/N2_) selectivities. A possible mechanism model revealing that the crosslinking degree played a key role in the gas separation process was also proposed.

The deteriorating environment caused by the global warming has become an immediate threat nowadays. Even though the excessive release of CO_2_ is considered as a real threat for the global warming it is also regarded as a new energy resource which is attracting much attention nowadays. The conventional techniques for capturing CO_2_ include “wet scrubbing” using alkaline solutions and membrane separation. However, “wet scrubbing” and traditional membrane separation process possess some drawbacks which include high cost, erosion of equipment and irreversible process^1^,^2^,^3^,^4^. Recently, fabrication of CO_2_-responsive materials is regarded as a new approach to solve such drawbacks^2^,^3^,^4^,^5^,^6^. Meanwhile, invention of novel membrane separation technology has always attracted great interests in CO_2_ capture from economic and environmental points of view due to its various advantages such as low energy cost, high-efficiency, high-stability, ease of fabrication^7^,^8^,^9^,^10^,^11^,^12^,^13^,^14^,^15^. Literature reveals, according to the theory of gas separation, that there are several kinds of membranes including inorganic membranes^16^, polymeric membranes^17^,^18^,^19^,^20^,^21^,^22^,^23^,^24^,^25^ and facilitated membranes^26^,^27^,^28^ which are used for CO_2_ capture^29^.

It was observed that the polymers containing EO (ethylene oxide) units were widely used in the separation of CO_2_ from other gases^30^,^31^,^32^,^33^,^34^,^35^,^36^. It was found that EO units showed strong interactions with CO_2_ compared with other gases such as N_2_, CH_4_, H_2_, especially with the temperature increased, EO units became more favored to CO_2_, which resulted in high CO_2_ permeabilities and selectivities over other gases^37^. A high content of EO segments was supposed to lead high CO_2_ permeabilities according to the reported principles^38^, however, it was explored that the PEO chains with high molecule weight was easy to crystallize, resulting in the decrease of the chain mobility, and hence exhibiting a reduction in the CO_2_ permeabilities and selectivities. Therefore, in order to avoid such crystallization, several methods were proposed, such as mixing the liquid PEO with rigid polymers^39^, doping PPO^40^, building a network by crosslinking PEO^37^,^41^,^42^,^43^,^44^,^45^,^46^,^47^ and so on. The influence of crosslinking on gas permeation has always been the issues researchers concerned about. Lin *et al*. have studied the effect of crosslinking on gas permeabilities in crosslinked Poly(Ethylene Glycol) Diacrylate, notably, they found that crosslinking had negligible effect on the gas separation properties of Poly(Ethylene Glycol) Diacrylate^39^. However, Our previous work reported that a kind of CO_2_-selective membrane consisting of EO segments and commercially available UV-crosslinker coumarin achieved high CO_2_ selectivities compared with N_2_ and He after UV-crosslinking, which suggested that the impact of crosslinking on gas permeation properties existed^48^. Based on this, it is our motivation in this work to have an insight on the role of crosslinking degree during the gas separation process of PEO-based membranes.

Chalcone was chosen by Iyoda and his coworkers as an UV-crosslinker to better control the crosslinking degree in their previous work^49^,^50^. It was because that the chalcone could be crosslinked like “head to head” and “head to tail” and hence forming a higher crosslinking degree of membrane structure as shown in Fig. 1. Therefore, in this work, in order to better understand the correlation of crosslinking degree and CO_2_ permeation properties, chalcone was selected as a unique crosslinkable segment instead of coumarin. In detail, chalcone was designed as a UV-crosslinker to study the regulation of gas permeation and crosslinking degree. For this, firstly, the chalcone segments and EO units were synthesized. Further, a series of block copolymers consisting of PEO (poly(ethylene oxide)) and PMA (poly(methacrylate) with chalcone mesogens) with different block ratios (PEO_11_-*b*-PMA(rChal)_7_, PEO_11_-*b*-PMA(rChal)_9_, PEO_11_-*b*-PMA(rChal)_12_, PEO_11_-*b*-PMA(rChal)_16_) were fabricated using Reversible Addition-Fragmentation Chain Transfer Polymerization (RAFT) method. The prepared copolymers were further investigated to examine the changes of gas permeabilities and selectivities of the membranes before and after UV crosslinking, respectively. Moreover, the variations of gas permeabilities and selectivities by changing the fraction of the crosslinkable block and crosslinking time were discussed. Based on the obtained results, a plausible mechanism between crosslinking degree and CO_2_ permeation properties was also demonstrated.

## Results and Discussion

### Characterizations of crosslinking degree

**Figure 2:**
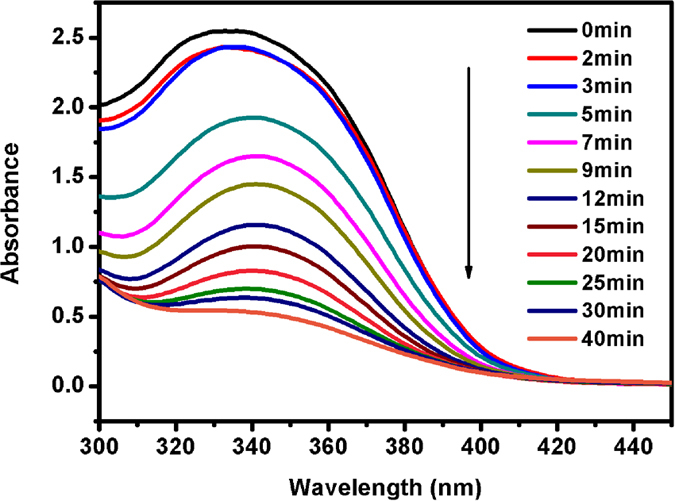
Photoreactions of the block copolymers with irradiation at 365 nm.

The effect of UV-crosslinking time on the prepared membranes was examined by UV-vis absorption spectroscopy. Figure 2 exhibited the typical UV-Vis spectra of the prepared membrane UV-crosslinking for various time intervals. The UV-Vis spectral trend was monitored at special chalcone absorption band appearing in the range of 300 to 450 nm. The observed UV-vis absorption spectroscopy revealed that the π-π* transition of the chalcone unit had a decrease from the 340 nm absorption band. The observed photoreaction could be explained according to the dimerization of the chalcone moieties through the [2 + 2] cyclization of the double bond^51^. Interestingly, it was observed that with increasing the UV-crosslinking time, the change of absorption spectra tended to balance, which illustrated that the greatest degree of crosslinking reached at 40 min.

#### Comparison of CO_2_ permeation properties of the membranes before and after crosslinking

**Figure 3:**
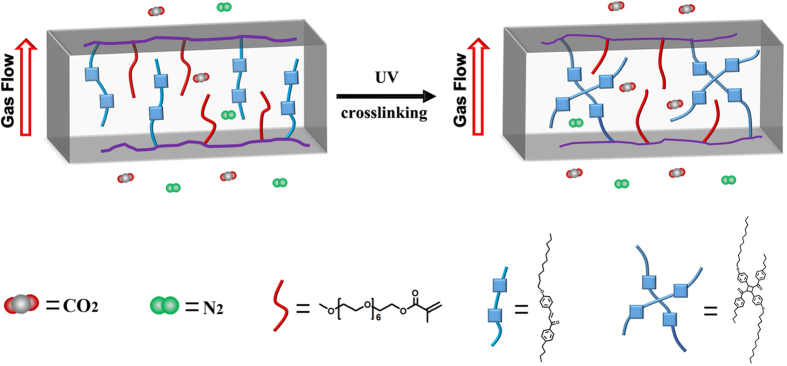
Graphical explanation of PEO-*b*-PMA(rChal) diblock copolymer thin film and CO_2_ separation from N_2_ gases (CO_2_/N_2_).

Table 1 showed the gases permeation and CO_2_/N_2_ selectivities of the un-crosslinked film compared with crosslinked film with PEO_11_-*b*-PMA(rChal)_7_ at different temperature. The α_CO2/N2_ data for other films are demonstrated in Tables S1 and S2. As shown in Table 1, the α_CO2/N2_ of these two membranes were increasing with rising the temperature. Interestingly, α_CO2/N2_ of un-crosslinked membrane was 2.76 at 60 °C which was much smaller than α_CO2/N2_ of crosslinked membrane equal to 12.56. (Figure S1) The selectivities was mainly related to solubility selectivities and diffusivity selectivities which mainly depended on the interactions of EO unit with CO_2_ and the free volume of EO, respectively. To explain this phenomenon, a probable mechanism model was proposed to interpret the increase of α_CO2/N2_ (Fig. 3). For un-crosslinked membrane, the EO units and the chalcone units had a large excess free volume and increased the mobility of the chain with the temperature rising, which led to higher gas permeabilities. Notably, differed from N_2_, the CO_2_ had a strong interaction with EO units, causing the PEO chains more flexible, which also contributed to the final α_CO2/N2_. Referred to crosslinked membrane, for the diffusivity selectivities, crosslinking structure limited the free volume and hence the EO units were confined in the hard regions of crosslinked chalcone walls. With increasing the temperature, the crosslinked hard regions were hardly moved but EO units were more flexible in the limited domain. However, for N_2_, the flexible EO units would lead to denser barriers in a limited area^48^, and then the less N_2_ molecules went through the free volume which produced a low gas permeabilities for N_2_. For CO_2_, the interactions between EO units and CO_2_ increased with rising the temperature and plasticized EO regions which lead to more flexible EO fraction, thus further increasing the CO_2_ permeabilities^52^. Therefore, with such aforementioned discussion, it can be concluded that the crosslinked membranes showed a favored gas selectivities towards CO_2_ with increasing the temperature.

#### Comparison of gas permeation properties of the membranes composed of various block ratio

**Figure 4:**
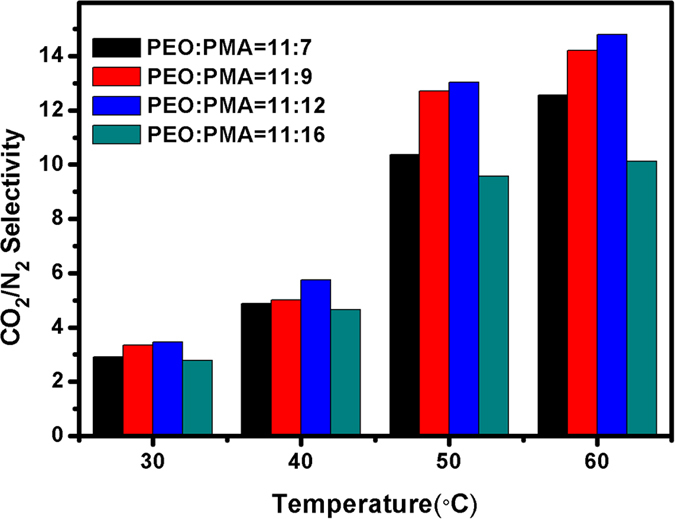
α_CO2/N2_ with PEO:PMA = 11:7(black), 11:9(red), 11:12(blue) and 11:16(green) at different temperature.

In this study, four kinds of block copolymers with different block ratios, i.e. PEO_11_-*b*-PMA(rChal)_7_, PEO_11_-*b*-PMA(rChal)_9_, PEO_*11*_-*b*-PMA(rChal)_12_, PEO_11_-*b*-PMA(rChal)_16_ were prepared and consequently, four membranes were exposed to UV light (40 min) for complete crosslinking. Table 2 showed the data of crosslinked membranes with the block ratios of PEO_11_-*b*-PMA(rChal)_9_, PEO_11_-*b*-PMA(rChal)_12_ and PEO_11_-*b*-PMA(rChal)_16_ at different temperatures. It was clearly indicated that all of these block copolymers possessed same tendency with the block ratio of PEO_11_-*b*-PMA(rChal)_7_. At 60 °C, α_CO2/N2_ reached to 12.56 when the mole percentage of PMA (PMA%) was about 39%. Further, the α_CO2/N2_ reached to 14.79 when PMA% was approximately equal to 50%, however, the α_CO2/N2_ dropped to 10.13 when PMA% was greater than 60% (Fig. 4).

**Figure 5:**
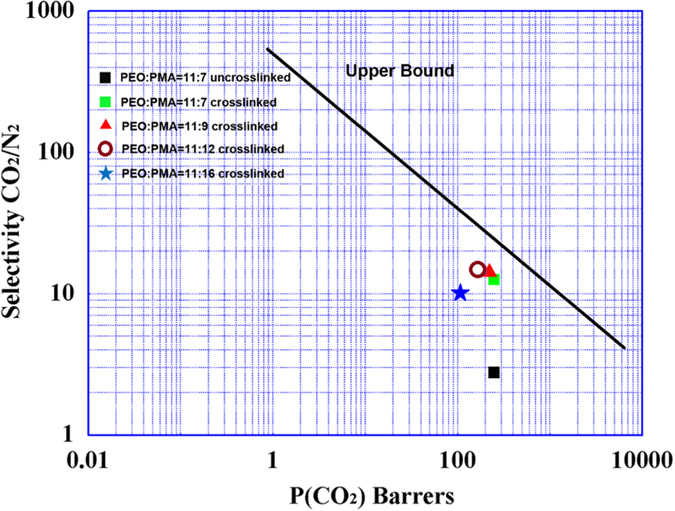
Robeson Upper Bound for CO_2_/N_2_ separation in 2008 with different block ratio copolymer membranes^53^.

For solubility selectivities, the interactions between EO and CO_2_ increased with rising the temperature. For diffusivity selectivities, the complicated crosslinking network led to the limited free volume. Moreover, crosslinking segments barely moved with the vary of temperature, so the N_2_ denser barriers increased with the more crosslinking units^48^. When the ratio of PEO:PMA varied from 11:7 to 11:12, the N_2_ permeabilities decreased as the data described. In contrast, CO_2_ plasticized EO chain to be more flexible which resulted in higher CO_2_/N_2_ selectivities. However, the continuous increase of mole percentage of chalcone segments did not represent sustainable rising trend in α_CO2/N2_ but form a more rigid crosslinking framework instead. The rigid framework structure limited the mobility of EO segments in a large degree, which became obstacles for CO_2_ transfer, representing a sharp reduction of α_CO2/N2_. As shown in Fig. 5, the highest CO_2_ selectivities of crosslinked membranes in this work was much closer to upper bound^53^ than un-crosslinked ones. Taking account of these factors, the content of crosslinking segments after fully crosslinking played a key role in CO_2_ gas permeable membrane.

### Comparison of CO_2_ permeation properties of a fixed block ratio BCP under different irradiation time

For this study, the PEO_11_-*b*-PMA(rChal)_12_ was treated as an example. (The data for other membranes are shown in Tables S3 and S4). Chalcone was an unique UV-crosslinker because of its easy crosslinking degree control by altering the UV irradiation time, which provided us a feasible way to verify the mechanism that crosslinking degree affected the ultimate CO_2_ permeation properties. Table 3 presented the system data obtained under different UV irradiation time 0 min, 5 min, 15 min and 40 min (fully crosslinked) at 30 °C and 60 °C, respectively. The observed results (Figure S2) revealed that the α_CO2/N2_ was rising with the variation of UV irradiation time. Specifically, the data of α_CO2/N2_ was 2.43 without UV irradiation at 60 °C and the α_CO2/N2_ reached to 6.46 after 5 min UV irradiation. Further, the α_CO2/N2_ value was reached to the maximum of 14.79 after 40 min of UV irradiation, which revealed the full crosslinking. With increasing the irradiation time, the crosslinking degree in chalcone units increased which were minimizing free volume of the framework, resulting in the decreasing of N_2_ permeabilities. Meanwhile, harder crosslinked PMA segments may also be contributed to the improvement of CO_2_/N_2_ selectivities. For diffusivity selectivities, CO_2_ would make EO chains more flexible in the limited area surrounded by the crosslinked wall, which caused high CO_2_ permeabilities. On the other hand, the confinement of free volume would result in denser barriers as explained above, leading to the low N_2_ permeabilities. Thus, based on the observed results, one can conclude that the demonstrated mechanism is fully consistent with the obtained results.

## Conclusion

In summary, we have successfully synthesized a series of diblock copolymers incorporating a novel UV-crosslinkable chalcone based on PEO chains using a facile RAFT process. Interestingly, it was observed that crosslinked membrane exhibited high CO_2_ permeabilities over N_2_ and hence showing high selectivities of CO_2_, in contrast with un-crosslinked membrane. Further, the detailed studies revealed that the diblock copolymers with different proportion displayed various selectivities. It was researched that the rising amounts of chalcone within certain limits enhanced the crosslinking degree by which the EO fractions become more flexible and thus exhibiting a higher CO_2_ permeabilities and selectivities with temperature enhancement. However, excess crosslinking chalcone fragment formed an ultra-rigid framework and confined the transfer of CO_2_ through the membrane, which resulted in low CO_2_ permeabilities. Thus, tunable CO_2_ selectivities could be achieved by monitoring the crosslinking degree of membranes. The presented work provided further applications of UV-crosslinking network for CO_2_ separation.

## Materials and Methods

### Materials

All the chemicals were analytical grade and used as received without any further purifications. 4-hydroxybenzaldehyde, Methoxypolyethylene glycols, 2-(Dodecyl- thiocarbonothioylthio)-2-methylpropionic acid (DDMAT), 11-bromoundecan-1-ol, Azo-bisisobutyronitrile (AIBN), Methacryloyl chloride, 4-butylphenylethylketone, N,N-Dimethylformamide (DMF), were all purchased from Sigma-Aldrich and Alfa-Aesar. Anisole was procured from Sigma-Aldrich with extra-dry grade purification.

### Measurements

The prepared materials were characterized in detail using several techniques. The ^1^H-NMR measurements were performed on Bruker AV-300 spectrometers in chloroform-d using tetramethylsilane (TMS; δ = 0) as internal reference. All copolymers were examined by gel permeation chromatography (Malvern, GPC 270) as reported in our previous work^48^. The standard sample of GPC is Polystyrene(PS) and Mn = 99385, the measured solvent was THF. The DSC curve was measured in DSC(NETZSCH, STA449F3). Gas permeation measurements were carried out in the similar manner as reported in the literature by the authors; i.e. a home-made stainless steel permeation apparatus as described previously^21^,^48^. The UV-crosslinking of the films was monitored by UV-Vis absorption spectroscopy. The average thickness of the tested film was examined by ellipsometry. Six sections on each membrane were measured respectively to calculate the average thickness of 1.8 ± 0.1 μm.

### Synthesis of the monomer chalcone

**Figure 6:**
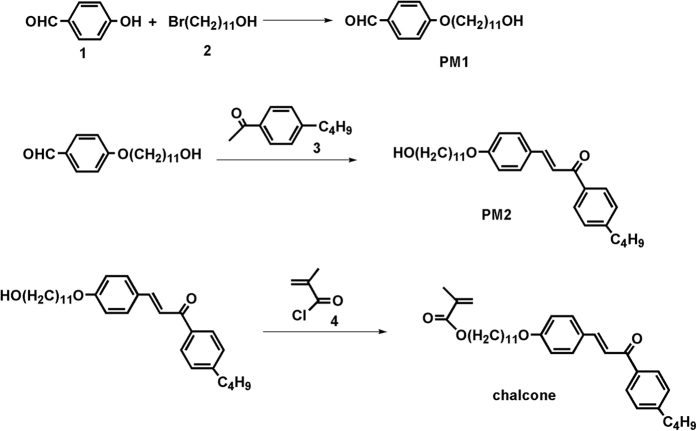
The synthesis route of chalcone.

The synthesis of monomer chalcone was done according to the Fig. 6 using organic synthetic procedure. The prepared material was purified with typical process and the purified product was characterized using ^1^H-NMR spectroscopy.

#### Preparation of PM1

To prepare the PM1, in a typical reaction process, 4-hydroxybenzaldehyde (15.27 g, 125 mmol), 11-bromoundecan-1-ol (32.66 g, 125 mmol) and DMF (100 mL) were added in a 3-necked flask under continuous stirring. The mixture was stirred until the materials were completely dissolved. Consequently, 1.88 g NaI and 34.56 g K_2_CO_3_ were added in the resultant solution and stirred again for 30 min. After stirring, the resultant mixture was reflux for 24 h. After desired reaction time, the reaction was terminated and the mixture was cooled at room-temperature and the solvent was removed using rotary evaporation process. Subsequently, the residue was added to water and thus twice extracted with dichloromethane (DCM). Finally, the organic layer was separated and dried over MgSO_4_ which was filtered. The obtained precipitate was then purified by column chromatography which finally provided a white solid (43.13 g). Yield: 90%. The ^1^H NMR data measured in deuterochloroform (CDCl_3_; 300 MHz) solvent exhibited several chemical shifts at δ 9.87 (1H, s), 7.84 (2H, d, J = 8.7 Hz), 6.99 (2H, d, J = 8.7 Hz), 4.03 (2H, t, J = 6.5 Hz), 3.64 (2H, t, J = 6.6 Hz), 1.81(2 H, m), 1.59–1.29 (12H, m). The typical ^1^H-NMR spectrum is shown in Figure S3. ^13^C-NMR (400 MHz, CDCl_3_, δ, ppm) 191.39, 164.26, 132.20, 129.73, 114.76, 77.15, 63.21, 32.92, 29.41, 26.28; TOF MS (C_18_H_28_O_3_) *m*/*z:* calcd. for 292.410, found 293.212.

#### Preparation of PM2

To prepare PM2, in a typical process, PM1 (34.9 g, 100 mmol) and 3 namely, 4-butylphenylethylketone (21 g, 100 mmol) were mixed in alcoholic sodium hydroxide (50 mL, 10%) solution under continuous stirring. After 12 h stirring at room-temperature, the reaction mixture was poured into ice-water. The solid precipitate was formed which was collected by filtration and dried. The dried product was the further purified by column chromatography and finally yellow solid was obtained (50.31 g). Yield: 90%. The ^1^H-NMR data measured in deuterochloroform (CDCl_3_; 300 MHz) solvent exhibited several chemical shifts at δ (ppm): 7.90–7.81 (d, 4 H, phenyl proton), 7.76 (d, 1H) 7.31–7.28 (d, 2H, phenyl proton), 7.19 (d, 1 H), 7.00–6.97 (m, 2H, phenyl proton), 4.11 (t, 2H, CH_2_O), 4.03 (t, 2H, CH_2_O), 2.68 (t, 2H, CH_2_Ph), 1.94 (s, 3H, CH_2_, d, C(CH3)-), 1.82 (m, 2H, CH2), 1.70–1.31 (m, 20H, (CH_2_)_10_), 0.94 (t, 3 H, -(CH_2_)_3_CH_3_) (Figure S4). ^13^C-NMR (400 MHz, CDCl_3_, δ, ppm) 190.32, 161.43, 148.23, 144.33, 136.37, 130.45, 128.72, 127.64, 120.00, 115.12, 77.17, 63.22, 35.71, 33.27, 32.81, 29.49, 25.75, 22.34, 13.90; TOF MS (C_30_H_42_O_3_) *m/z:* calcd. for 453.310, found 453.227.

#### Preparation of chalcone

To prepare the chalcone, in a typical reaction process, PM2 (42.8 g, 115 mmol), TEA (1.82 ml, 120 mmol) and 100 ml DCM were added in a dry round-bottom flask. Consequently, methacryloyl chloride (14.57 ml, 115 mmol) was added dropwise to the mixture at 0 °C and the reaction was continued overnight. The obtained product was then added to the water and subsequently extracted with DCM twice. The organic layer was then separated and dried over MgSO_4_. Finally, the obtained product was filtered and purified by column chromatography which gives a light yellow solid (40.66 g). Yield: ~95%. The ^1^H-NMR data measured in deuterochloroform (CDCl_3_; 300 MHz) solvent exhibited several chemical shifts at δ (ppm): 7.90–7.81 (d, 4H, phenyl proton), 7.76 (d, 1H), 7.31–7.28 (d, 2H, phenyl proton), 7.19 (d, v1H), 7.00–6.97 (m, 2H, phenyl proton), 6.10 (s, 1H, (H=C)), 5.54 (s, 1H, H=C)), 4.11 (t, 2H, CH_2_O), 4.03 (t, 2H, CH_2_O), 2.68 (t, 2H, CH_2_Ph), 1.94 (s, 3H, CH_2_, d, C(CH_3_)-), 1.82 (m, 2H, CH_2_), 1.70–1.31 (m, 20H, (CH_2_)_10_), 0.94 (t, 3H, -(CH_2_)_3_CH_3_) (Figure S5). ^13^C-NMR (400 MHz, CDCl_3_, δ, ppm) 190.32, 167.66, 161.43, 148.25, 144.30, 136.28, 130.14, 128.62, 127.63, 125.08, 119.80, 114.84, 77.00, 68.30, 35.71, 33.33, 29.57, 25.97, 22.56, 18.31, 13.89; TOF MS (C_34_H_46_O_4_) *m/z:* calcd. for 518.630, found 519.281.

#### Preparation of EO precursor

**Figure 7:**
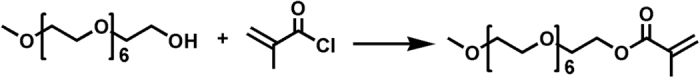
The synthesis route of EO precursor.

The synthesis of monomer was done according to the Fig. 7. In a typical synthesis process, solution of 10.5 g (100 mmol) methacryloyl chloride, in 30 mL DCM, was dropwise added to a mixture of 15.1 g (150 mmol) triethylamine and 35 g (100 mmol) methoxy polyethylene glycols (Mn = 350) in 100 mL of DCM at 0 °C. The resultant mixture was then stirred overnight. After overnight stirring, the resultant mixture was filtered and the obtained crude product was added to 100 ml water and subsequently extracted with DCM twice. Finally, the solvent was dried by rotary evaporation which leaves oily liquid (43.23 g). Yield: 95%. The ^1^H-NMR data measured in deuterochloroform (CDCl_3_; 300 MHz) solvent exhibited several chemical shifts at δ (ppm):1.93 (s, 3H), 3.24 (s, 3H), 3.54 (t, 24H, CH_2_CH_2_O), 3.65 (t, 2H), 4.32 (t, 2H), 5.58 (s, 1H, -C(CH_3_)=CH_2_), 6.15 (s, 1H, -C(CH_3_)=CH_2_).(Figure S6); ^13^C-NMR (400 MHz, CDCl_3_, δ, ppm) 167.32, 136.34, 125.56, 76.85, 70.50, 69.20, 63.55, 59.39, 18.31; TOF MS (C_20_H_38_O_9_) *m/z:* calcd. for 422.510; found 422.277.

#### Preparation of PEO Macro-initiators

**Figure 8:**
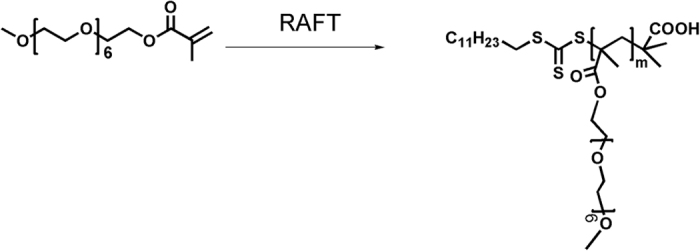
The synthesis route of PEO macro-initiators.

The macro-initiator was synthesized by the Reversible Addition-Fragmentation Chain Transfer Polymerization (RAFT) method. In a typical reaction process, 2.0 g (4.8 mmol) of EO precursor, 34.94 mg (0.096 mmol) of 2-(Dodecylthio-carbon-thioyl-thio)-2-methyl propionic acid (DDMAT) and 3.149 mg (0.0192 mmol) of Azobisisobutyro-nitrile (AIBN) were mixed in a 10 mL Shreck bottle with 1.5 ml anhydrous anisole. Consequently, the resultant mixture was degassed four times using the freeze pump-thaw procedure and the bottle was sealed under vacuum. The sealed bottle was then placed in a preheated oil bath (90 °C) for 12 h. Finally, the solution was precipitated in hexane (Fig. 8). The obtained yield was 45.5% (0.91 mg). The observed Mn and PDI are 4600 and 1.13, respectively. The ^1^H-NMR, measured in deuterochloroform, exhibited several chemical shifts at δ (ppm):1.25–1.34 (t, 23H, -C_12_H_23_), 3.36–3.42 (s, 3H, -OCH_3_) (Figure S7); ^13^C-NMR (400 MHz, CDCl_3_, δ, ppm) 216.01, 177.26, 174.59, 70.65, 68.34, 65.25, 52.46, 46.03, 43.37, 35.82, 33.37, 29.73, 29.62, 29.50, 29.27, 28.25, 26.23, 22.36, 13.94.

### Preparation of PEO-*b*-PMA(rChal) diblock copolymers

**Figure 9:**
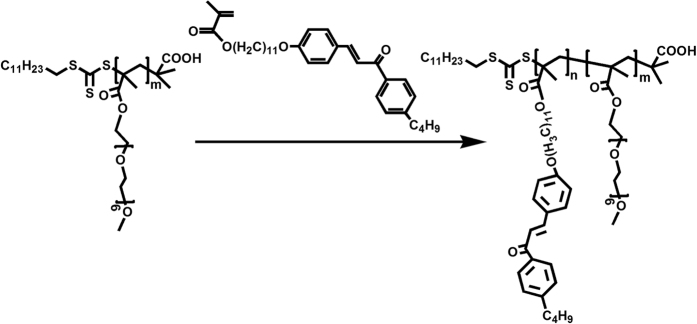
The synthesis route of PEO-*b*-PMA(rChal) (11:7, 11:9, 11:12, 11:16) diblock copolymers.

**Figure 10:**
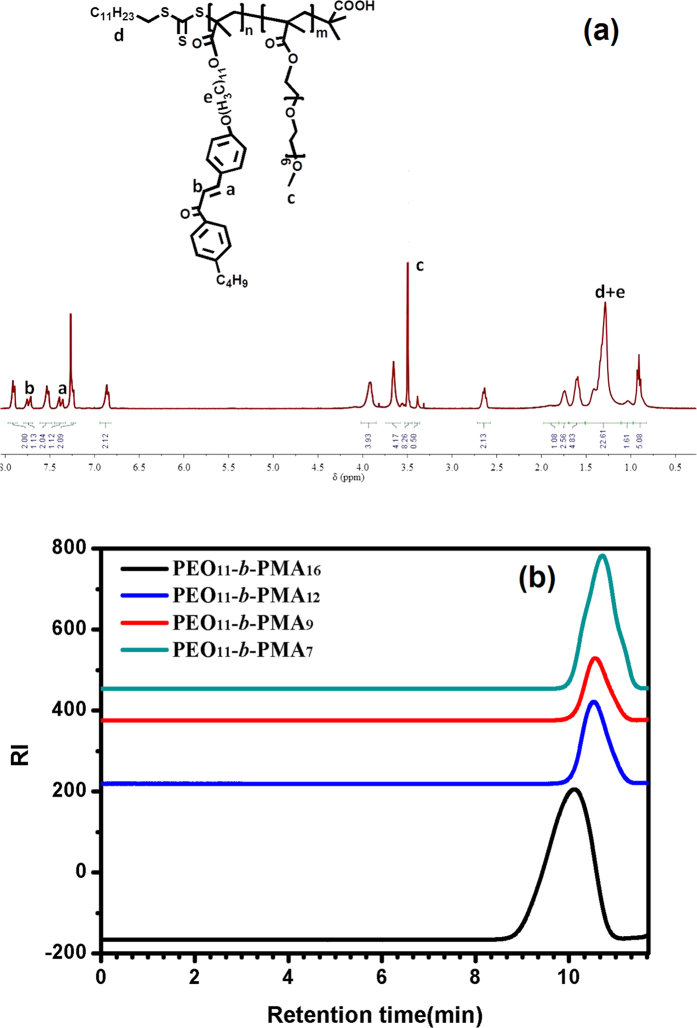
The ^1^H-NMR and GPC of different block ratios of diblock copolymers.

A series of PEO-*b*-PMA(rChal) containing a chalcone mesogen with different content of polymerization were synthesized by RAFT method. The targeted material was prepared as presented in Fig. 9. As an example, a procedure to prepare PEO_11_-*b*-PMA(rChal)_7_ is described here. In a typical reaction process, 0.097 g (1 eqv) PEO macro-initiators, 0.24 g (30 eqv) chalcone and 0.0003 g (0.12 eqv) AIBN were mixed in a 10 mL Shreck bottle with 1.8 ml anhydrous anisole. Then, the mixture was degassed four times using the freeze pump-thaw procedure and sealed under vacuum. The sealed bottle was then placed in a preheated oil bath (90 °C) for 17 h. The solution was precipitated in diethyl ether and finally pure diblock copolymer was obtained. Figure 10 showed the typical ^1^H-NMR (a) and GPC (b) results. The observed Mn and PDI of PEO_11_-*b*-PMA(rChal)_7_, PEO_11_-*b*-PMA(rChal)_9_, PEO_11_-*b*-PMA(rChal)_12_, PEO_11_-*b*-PMA(rChal)_16_ are 8200 and 1.13, 9200 and 1.11, 10800 and 1.16, 13200 and 1.19. The ^1^H-NMR data measured in deuterochloroform (CDCl_3_; 300 MHz) solvent exhibited several chemical shifts at δ (ppm):1.30–1.46 (t, 23H, -C_12_H_23_), 3.51–3.57 (s, 3H, -OCH_3_), 1.19–1.23 (s, 22H, -O(CH_2_)_11_O-); ^13^C-NMR (400 MHz, CDCl_3_, δ, ppm) 208.61, 189.94, 177.90, 169.84, 164.89, 162.11, 148.52, 144.46, 136.15, 130.23, 128.69, 127.78, 115.09, 70.80, 68.31, 65.17, 35.77, 33.33, 29.63, 28.35, 26.18, 22.42, 14.13. The DSC curve of un-crosslinked block copolymer PEO_11_-*b*-PMA(rChal)_12_ was measured at a rate of 2 °C/min from 40 °C to 140 °C and shown in Figure S8.

### Preparation of thin membranes

The diblock copolymer membrane was made by spin-coating (1000 rpm) of 6 wt% chloroform solutions on Anodic Alumina Oxide (AAO) substrate. The prepared diblock copolymer membrane was placed in vacuum for 4 h at room-temperature. Consequently, the crosslinking of the block copolymer membrane was exposed to UV light (365 nm) for a desirable time and finally the UV-crosslinked membrane was obtained.

### Gas permeation measurements

A home-built gas permeation measurement system was used to estimate the gas permeation as described in our previous work^22^,^48^. The pure gases with different kinetic diameters such as N_2_ and CO_2_ were studied in this work. For this, a spin-coated membranes on AAO substrates were placed in the permeation cell with a support screen. The surface area of the tested membrane, available for gas transport, was estimated and found to be 2.84 cm^2^. The membranes were placed in the test system cell to investigate the gas permeation test. The gases, after passing through the membrane in the cell, was directed into a glass U-tube flow meter (Acol = 0.03 cm^2^) to give the volumetric flow rate of the gas. It was measured by recording the time (t) that was required for a liquid column to travel a distance (X_column_ = 10 cm). All the measurements were taken at ambient temperature and the values were obtained at steady-state (usually last for at least 2 h). The values were obtained from 10 independent measurements and the mean value and standard deviations were determined. The error in each case was <5%. The same experimental procedure was repeated for other targeted gas. In general, the permeation properties were sequentially measured for He, N_2_ and CO_2_, respectively. The permeance (P; 10^6^ cm·s^−1^·cmHg^−1^) was calculated based on the equation (1)^48^:


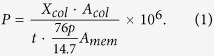


And the selectivities (*α*) of gas A, over gas B, was defined based on the below equation (2)^48^:


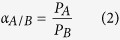


## Additional Information

**How to cite this article**: Li, J. *et al*. Probe Into the Influence of Crosslinking on CO_2_ Permeation of Membranes. *Sci. Rep.* **7**, 40082; doi: 10.1038/srep40082 (2017).

**Publisher's note:** Springer Nature remains neutral with regard to jurisdictional claims in published maps and institutional affiliations.

## Supplementary Material

Supplementary Information

## Figures and Tables

**Figure 1:**
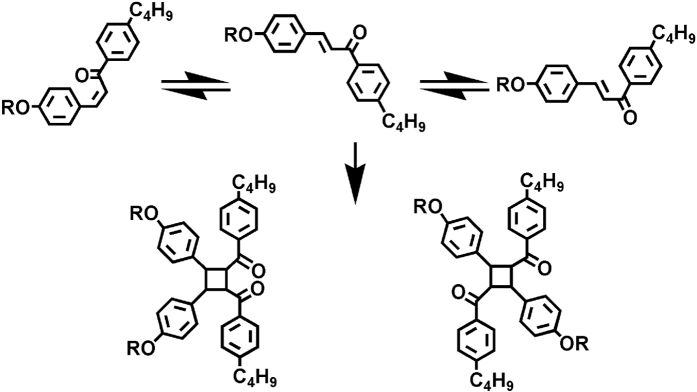
Photochemical Reaction of the chalcone under UV irradiation forming “Head to Head” and “Head to Tail” structures^49^.

**Table 1:** Pure Gas Permeabilities and Selectivities of PEO-*b*-PMA (rChal) (11:7) Un-crosslinked Compared with Crosslinked Membrane.

Temp (°C)	Condition	N_2_ Permeabilities (barrer)	CO_2_ Permeabilities (barrer)	α_(CO2/N2)_
30	Un-crosslinked	84.40	138.01	1.63
	Crosslinked	38.71	113.18	2.92
40	Un-crosslinked	85.16	178.91	2.10
	Crosslinked	31.79	155.30	4.89
50	Un-crosslinked	88.01	229.14	2.61
	Crosslinked	20.97	217.29	10.36
60	Un-crosslinked	90.67	250.46	2.76
	Crosslinked	19.89	249.87	12.56

Permeances at 1 × 10^6^ (cm·s^−1^·cmHg^−1^), were calculated by dividing the observed flow rate by the area of the membrane (2.84 cm^2^) and the pressure gradient (10 psi) employed, using porous Al_2_O_3_ membrane supports. The values were obtained from 10 independent measurements and the mean value and standard deviations were determined. The error in each case was <5%. The membrane PEO-*b*-PMA (rChal) showed no difference of gas permeation in humid environment.

**Table 2:** Pure Gas Permeabilities and Selectivities of Different Block Ratio PEO-*b*-PMA (rChal) Crosslinked Membrane.

Temp. (°C)	Block ratio (PEO:PMA)	N_2_ Permeabilities (barrer)	CO_2_ Permeabilities (barrer)	α_(CO2/N2)_
30	11:7	38.71	113.18	2.92
	11:9	32.43	108.65	3.35
	11:12	29.05	100.53	3.46
	11:16	26.13	72.62	2.78
40	11:7	31.79	155.30	4.89
	11:9	28.42	142.34	5.01
	11:12	21.40	123.35	5.76
	11:16	19.23	89.43	4.65
50	11:7	20.97	217.29	10.36
	11:9	17.01	216.41	12.72
	11:12	12.64	164.82	13.04
	11:16	11.15	106.71	9.57
60	11:7	19.89	249.87	12.56
	11:9	15.49	219.94	14.20
	11:12	11.19	165.69	14.79
	11:16	10.56	106.99	10.13

Permeances at 1 × 10^6^ (cm·s^−1^·cmHg^−1^), were calculated by dividing the observed flow rate by the area of the membrane (2.84 cm^2^) and the pressure gradient (10 psi) employed, using porous Al_2_O_3_ membrane supports. The values were obtained from 10 independent measurements and the mean value and standard deviations were determined. The error in each case was <5%. The membrane PEO-*b*-PMA (rChal) showed no difference of gas permeation in humid environment.

**Table 3:** Pure Gas Permeabilities and Selectivities of PEO-*b*-PMA(rChal) (11:12) Membrane under Different Crosslink Time.

Temp(°C)	Crosslink Time (min)	N_2_ Permeabilities (barrer)	CO_2_ Permeabilities (barrer)	α_(CO2/N2)_
30	0	80.43	125.48	1.56
	5	46.24	119.31	2.58
	15	35.61	108.24	3.04
	40	29.05	100.53	3.46
60	0	97.78	237.62	2.43
	5	31.62	204.34	6.46
	15	17.21	176.84	10.28
	40	11.19	165.69	14.79

Permeances at 1 × 10^6^ (cm·s^−1^·cmHg^−1^), were calculated by dividing the observed flow rate by the area of the membrane (2.84 cm^2^) and the pressure gradient (10 psi) employed, using porous Al_2_O_3_ membrane supports. The values were obtained from 10 independent measurements and the mean value and standard deviations were determined. The error in each case was <5%. The membrane PEO-*b*-PMA (rChal) showed no difference of gas permeation in humid environment.
