# Supplementary material for: Probe Into the Influence of Crosslinking on CO2 Permeation of Membranes
Source: Sci Rep. 2017 Jan 4;7:40082. doi: 10.1038/srep40082 (PMC5209686; doi:10.1038/srep40082)
Supplement: Supplementary Information [file srep40082-s1.doc]

**Supporting Information**

**Probe Into the Influence of Crosslinking on CO_2_ Permeation of Membranes**

**Jinghui Li,^1^ Zhuo Chen,^1^ Ahmad Umar,^2^ Yang Liu,^3^ Ying Shang,^1^ Xiaokai Zhang,^1^ Yao Wang^1,*^**

*^1^School of Chemistry and Environment, Key Laboratory of Bio-Inspired Smart Interfacial Science and Technology of Ministry of Education, Beihang University, Beijing 100191, PR China.*

*^2^Department of Chemistry, Faculty of Science and Arts and Promising Centre for Sensors and Electronic Devices (PCSED), Najran University, Najran 11001, Kingdom of Saudi Arabia.*

*^3^Beijing Key Laboratory of Radiation Advanced Materials, Beijing Research Center for Radiation Application, Beijing 100015, China.*

***Author to whom correspondence should be address:**

**Prof. Yao Wang**

School of Chemistry and Environment
Beihang University (Beijing University of Aeronautics & Astronautics) 
XueYuan Road No.37, HaiDian District
Beijing, China 100191
Phone (office): 86-10-8231-7801
Email: [yao@buaa.edu.cn](mailto:yao@buaa.edu.cn)

**Supplementary Tables**

**Table S1. Pure Gas Permeabilities and Selectivities of PEO-*b*-PMA(rChal)(11:9) Un-crosslinked compared with Crosslinked Membrane**

| **Temp (^o^C)** | **Condition** | **He**  **permeabilities**  **(barrer)** | **N_2_**  **permeabilities**  **(barrer)** | **CO_2_**  **permeabilities**  **(barrer)** | **CO_2_/N_2_**  **Selectivities** | **He/CO_2_**  **Selectivities** | **He/N_2_**  **Selectivities** |
| --- | --- | --- | --- | --- | --- | --- | --- |
| **30** | Un-crosslinked | 559.57 | 81.52 | 137.25 | 1.68 | 4.08 | 6.86 |
|  | Crosslinked | 436.68 | 32.43 | 108.65 | 3.35 | 3.99 | 13.39 |
|  |  |  |  |  |  |  |  |
| **40** | Un-crosslinked | 562.38 | 82.09 | 176.38 | 2.15 | 3.19 | 6.85 |
|  | Crosslinked | 415.69 | 28.42 | 142.34 | 5.01 | 2.92 | 14.63 |
|  |  |  |  |  |  |  |  |
| **50** | Un-crosslinked | 581.52 | 85.21 | 228.17 | 2.68 | 2.55 | 6.82 |
|  | Crosslinked | 378.46 | 17.01 | 216.41 | 12.72 | 1.75 | 22.25 |
|  |  |  |  |  |  |  |  |
| **60** | Un-crosslinked | 584.38 | 89.99 | 253.30 | 2.81 | 2.31 | 6.49 |
|  | Crosslinked | 370.13 | 15.49 | 219.94 | 14.20 | 1.68 | 23.89 |

Permeances at 1×10^6^ (cm·s^-1^·cmHg^-1^), were calculated by dividing the observed flow rate by the area of the membrane (2.84 cm^2^) and the pressure gradient (10 psi) employed, using porous Al_2_O_3_ membrane supports. The values were obtained from 10 independent measurements and the mean value and standard deviations were determined. The error in each case was <5%. The membrane PEO-*b*-PMA(rChal) showed no difference of gas permeation in humid environment.

**Table S2. Pure Gas Permeabilities and Selectivities of PEO-*b*-PMA(rChal)(11:12) Un-crosslinked compared with Crosslinked Membrane**

| **Temp (^o^C)** | **Condition** | **He**  **permeabilities**  **(barrer)** | **N_2_**  **permeabilities**  **(barrer)** | **CO_2_**  **permeabilities**  **(barrer)** | **CO_2_/N_2_**  **Selectivities** | **He/CO_2_**  **Selectivities** | **He/N_2_**  **Selectivities** |
| --- | --- | --- | --- | --- | --- | --- | --- |
| **30** | Un-crosslinked | 515.69 | 80.43 | 125.48 | 1.56 | 4.11 | 6.41 |
|  | Crosslinked | 406.17 | 29.05 | 100.53 | 3.46 | 4.03 | 13.98 |
|  |  |  |  |  |  |  |  |
| **40** | Un-crosslinked | 537.32 | 86.28 | 164.83 | 1.91 | 3.26 | 6.23 |
|  | Crosslinked | 320.83 | 21.40 | 123.35 | 5.76 | 2.60 | 14.99 |
|  |  |  |  |  |  |  |  |
| **50** | Un-crosslinked | 555.52 | 92.06 | 208.04 | 2.26 | 2.67 | 6.03 |
|  | Crosslinked | 286.76 | 12.64 | 164.82 | 13.04 | 1.74 | 22.25 |
|  |  |  |  |  |  |  |  |
| **60** | Un-crosslinked | 574.20 | 97.78 | 237.62 | 2.43 | 2.42 | 5.87 |
|  | Crosslinked | 276.14 | 11.19 | 165.69 | 14.79 | 1.66 | 24.66 |

Permeances at 1×10^6^ (cm·s^-1^·cmHg^-1^), were calculated by dividing the observed flow rate by the area of the membrane (2.84 cm^2^) and the pressure gradient (10 psi) employed, using porous Al_2_O_3_ membrane supports. The values were obtained from 10 independent measurements and the mean value and standard deviations were determined. The error in each case was <5%. The membrane PEO-*b*-PMA(rChal) showed no difference of gas permeation in humid environment.

**Table S3.** Pure Gas Permeabilities and Selectivities of PEO-*b*-PMA(rChal) (11:7) Membrane under Different Crosslink Time

| **Temp**  **(^o^C)** | **Crosslink time**  **(min)** | **He**  **permeabilities**  **(barrer)** | **N_2_**  **permeabilities**  **(barrer)** | **CO_2_**  **permeabilities**  **(barrer)** | **CO_2_/N_2_**  **Selectivities** | **He/CO_2_**  **Selectivities** | **He/N_2_**  **Selectivities** |
| --- | --- | --- | --- | --- | --- | --- | --- |
| **30** | 0 | 563.96 | 84.40 | 138.01 | 1.63 | 4.08 | 6.68 |
|  | 5 | 523.15 | 63.57 | 124.59 | 1.96 | 4.19 | 8.23 |
|  | 15 | 499.00 | 47.71 | 118.81 | 2.49 | 4.20 | 10.45 |
|  | 40 | 480.42 | 38.71 | 113.18 | 2.92 | 4.24 | 12.4 |
|  |  |  |  |  |  |  |  |
| **60** | 0 | 589.41 | 90.67 | 250.46 | 2.76 | 2.35 | 6.50 |
|  | 5 | 514.39 | 55.33 | 250.25 | 4.52 | 2.06 | 9.30 |
|  | 15 | 481.19 | 28.07 | 250.09 | 8.91 | 1.92 | 17.14 |
|  | 40 | 418.89 | 19.89 | 249.87 | 12.56 | 1.66 | 20.96 |

Permeances at 1×10^6^ (cm·s^-1^·cmHg^-1^), were calculated by dividing the observed flow rate by the area of the membrane (2.84 cm^2^) and the pressure gradient (10 psi) employed, using porous Al_2_O_3_ membrane supports. The values were obtained from 10 independent measurements and the mean value and standard deviations were determined. The error in each case was <5%. The membrane PEO-*b*-PMA(rChal) showed no difference of gas permeation in humid environment.

**Table S4.** Pure Gas Permeabilities and Selectivities of PEO-*b*-PMA(rChal) (11:9) Membrane under Different Crosslink Time

| **Temp**  **(^o^C)** | **Crosslink**  **time**  **(min)** | **He**  **permeabilities**  **(barrer)** | **N_2_**  **permeabilities**  **(barrer)** | **CO_2_**  **permeabilities**  **(barrer)** | **CO_2_/N_2_**  **Selectivities** | **He/CO_2_**  **Selectivities** | **He/N_2_**  **Selectivities** |
| --- | --- | --- | --- | --- | --- | --- | --- |
| **30** | 0 | 559.57 | 81.52 | 137.25 | 1.68 | 4.08 | 6.86 |
|  | 5 | 498.11 | 54.33 | 126.04 | 2.32 | 3.95 | 9.17 |
|  | 15 | 479.31 | 39.98 | 118.35 | 2.96 | 4.05 | 11.98 |
|  | 40 | 436.68 | 32.43 | 108.65 | 3.35 | 3.99 | 13.39 |
|  |  |  |  |  |  |  |  |
| **60** | 0 | 584.38 | 89.99 | 253.30 | 2.81 | 2.31 | 6.49 |
|  | 5 | 479.03 | 45.07 | 247.02 | 5.48 | 1.94 | 10.63 |
|  | 15 | 418.77 | 26.24 | 225.69 | 8.57 | 1.86 | 15.95 |
|  | 40 | 370.13 | 15.49 | 219.94 | 14.20 | 1.68 | 23.89 |

Permeances at 1×10^6^ (cm·s^-1^·cmHg^-1^), were calculated by dividing the observed flow rate by the area of the membrane (2.84 cm^2^) and the pressure gradient (10 psi) employed, using porous Al_2_O_3_ membrane supports. The values were obtained from 10 independent measurements and the mean value and standard deviations were determined. The error in each case was <5%. The membrane PEO-*b*-PMA(rChal) showed no difference of gas permeation in humid environment.

**Supplementary Figures**


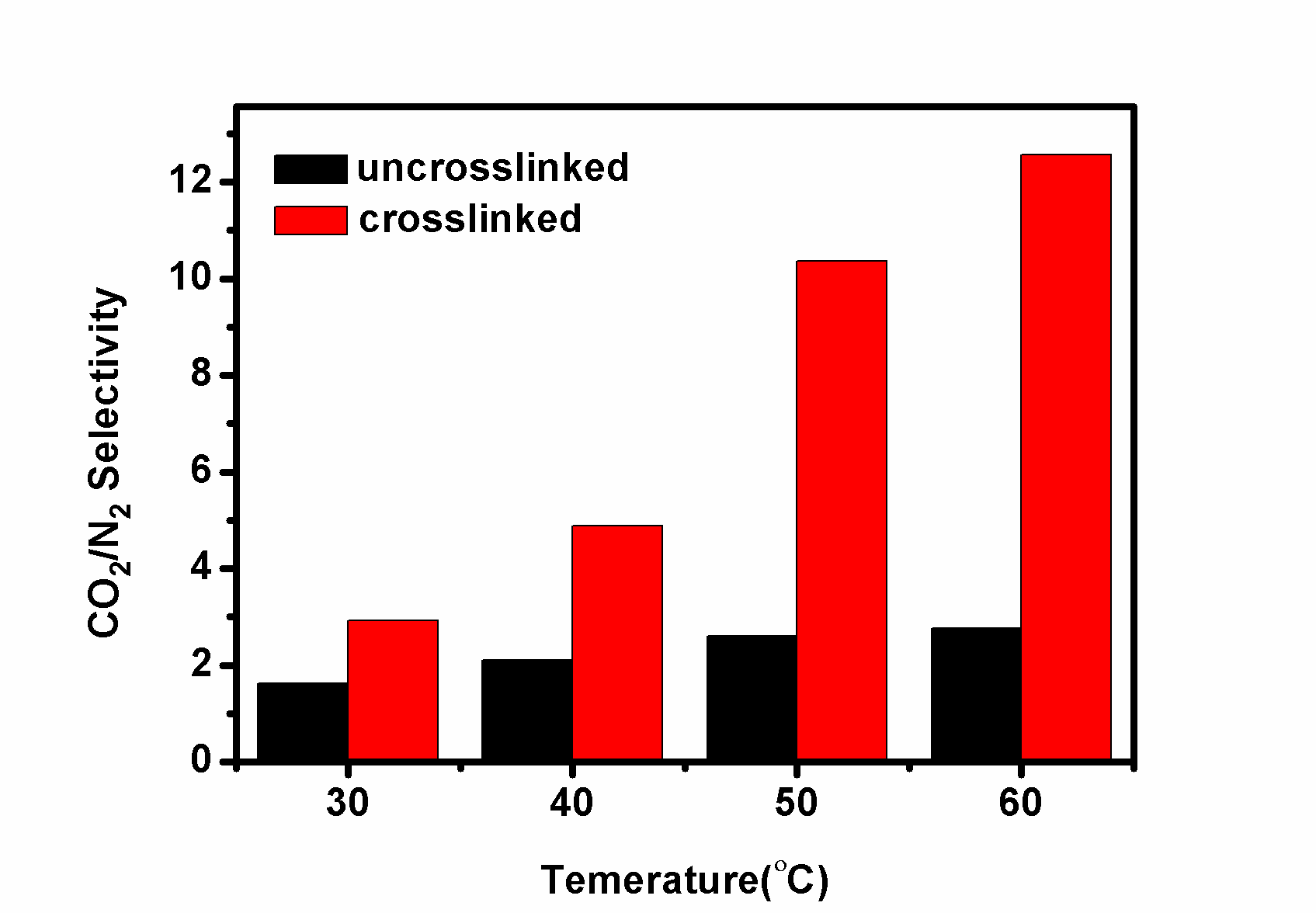


**Figure S1** The selectivities of CO_2_ and N_2_ before(black), after(red) crosslinking with PEO:PMA=11:7.

***
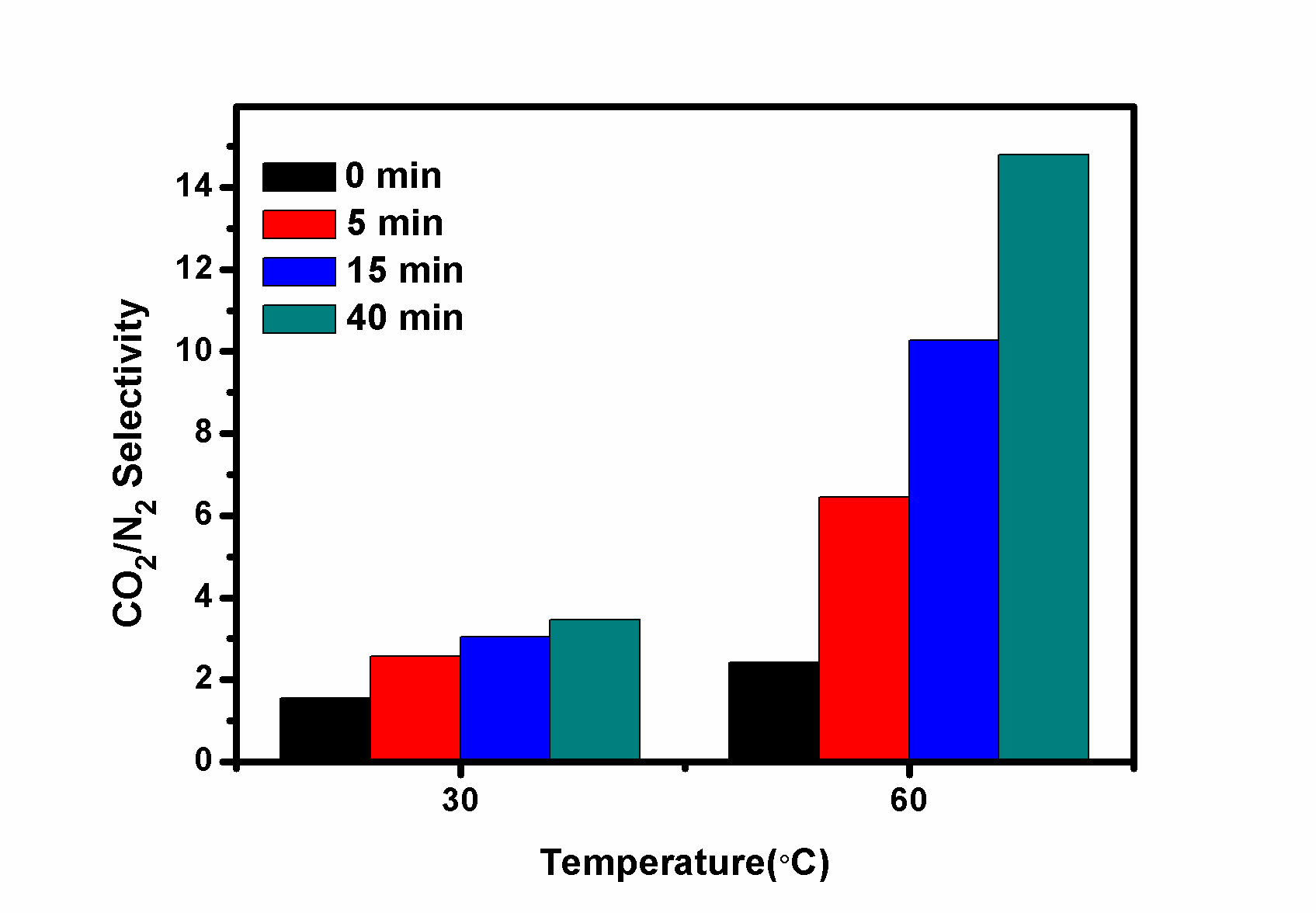
***

**Figure S2.** The selectivities of CO_2_ and N_2_ with PEO:PMA=11:12 under different crosslink time: 0min, 5min, 15min, 40min.


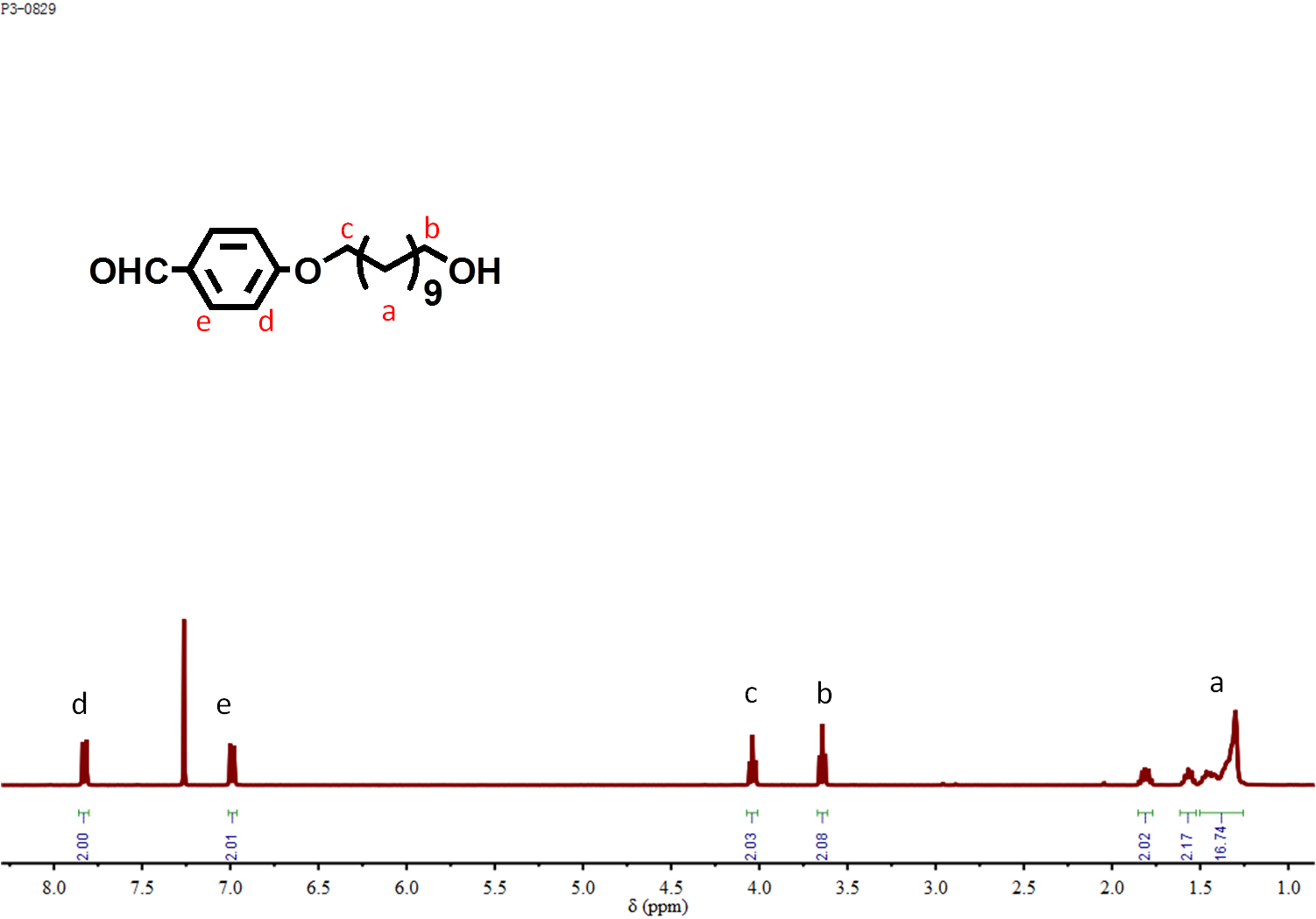


**Figure S3.** The ^1^H-NMR of PM1


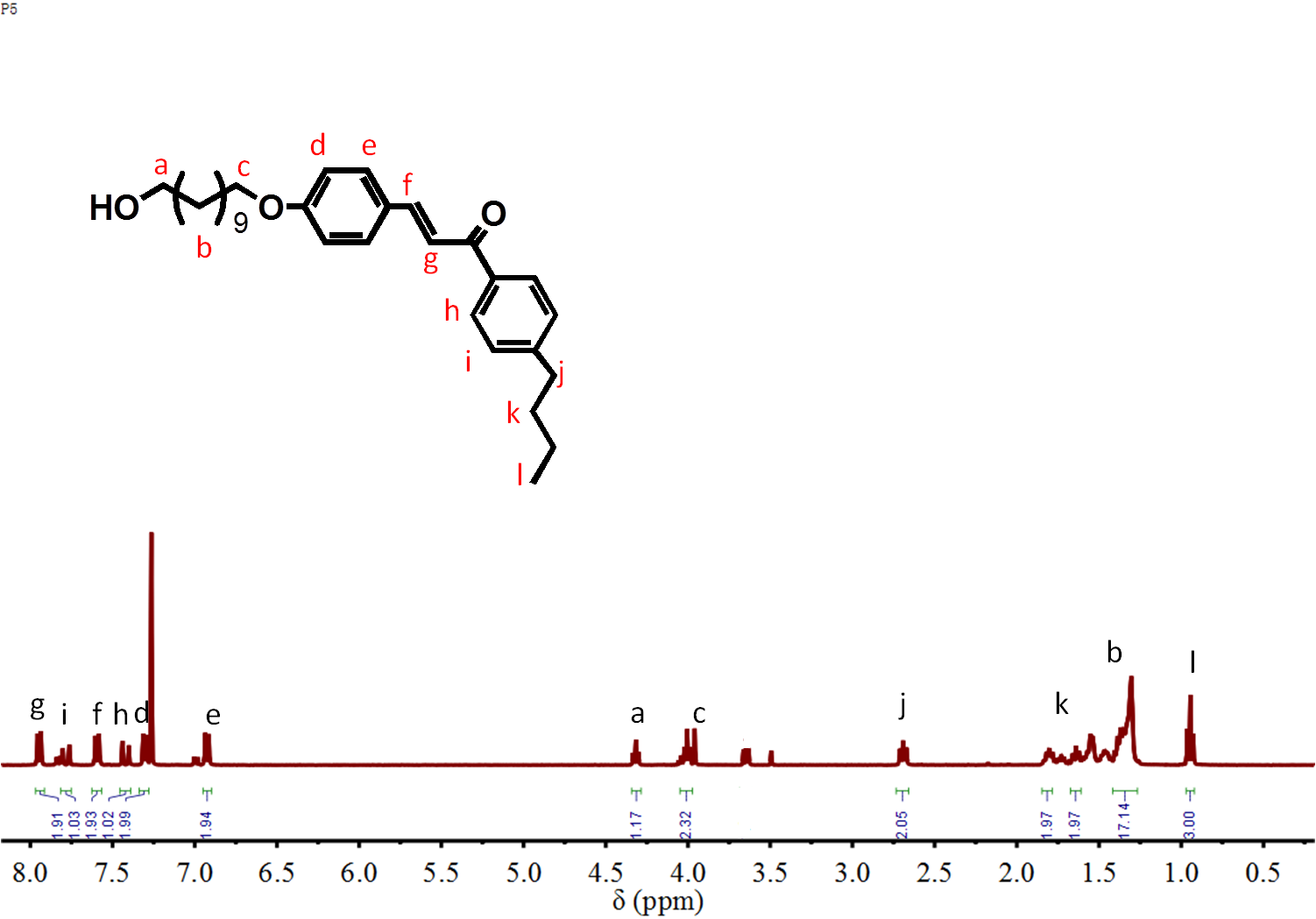


**Figure S4.** The ^1^H-NMR of PM2


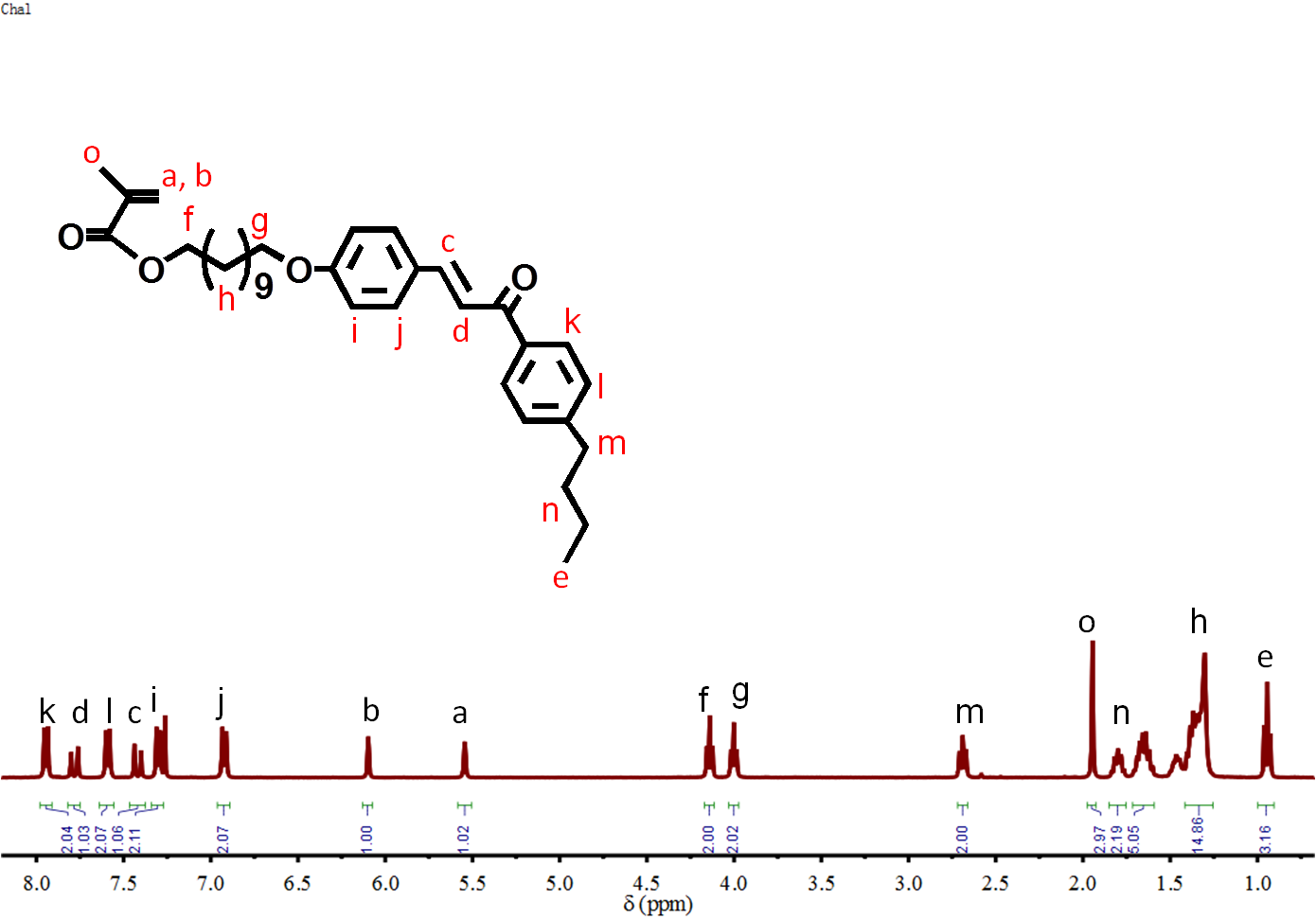


**Figure S5.** The ^1^H-NMR of chalcone


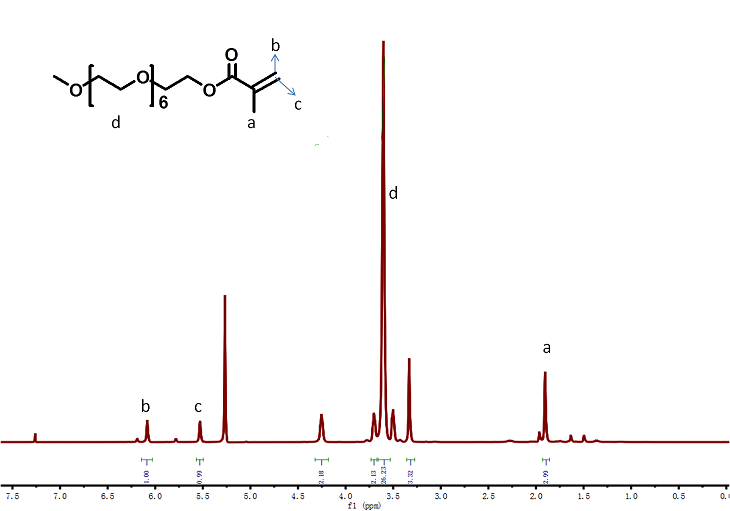


**Figure S6.** The ^1^H-NMR of EO precursor


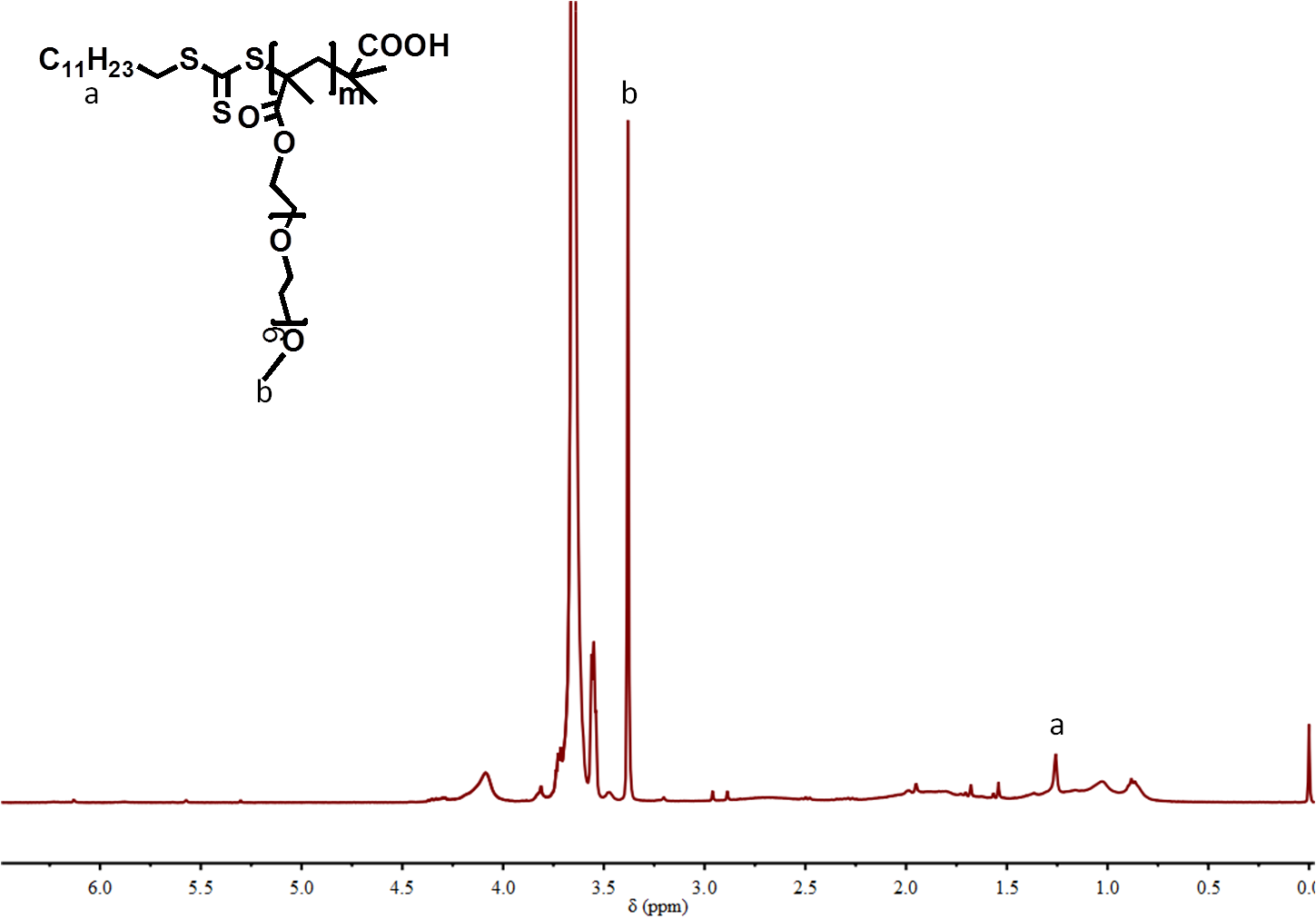

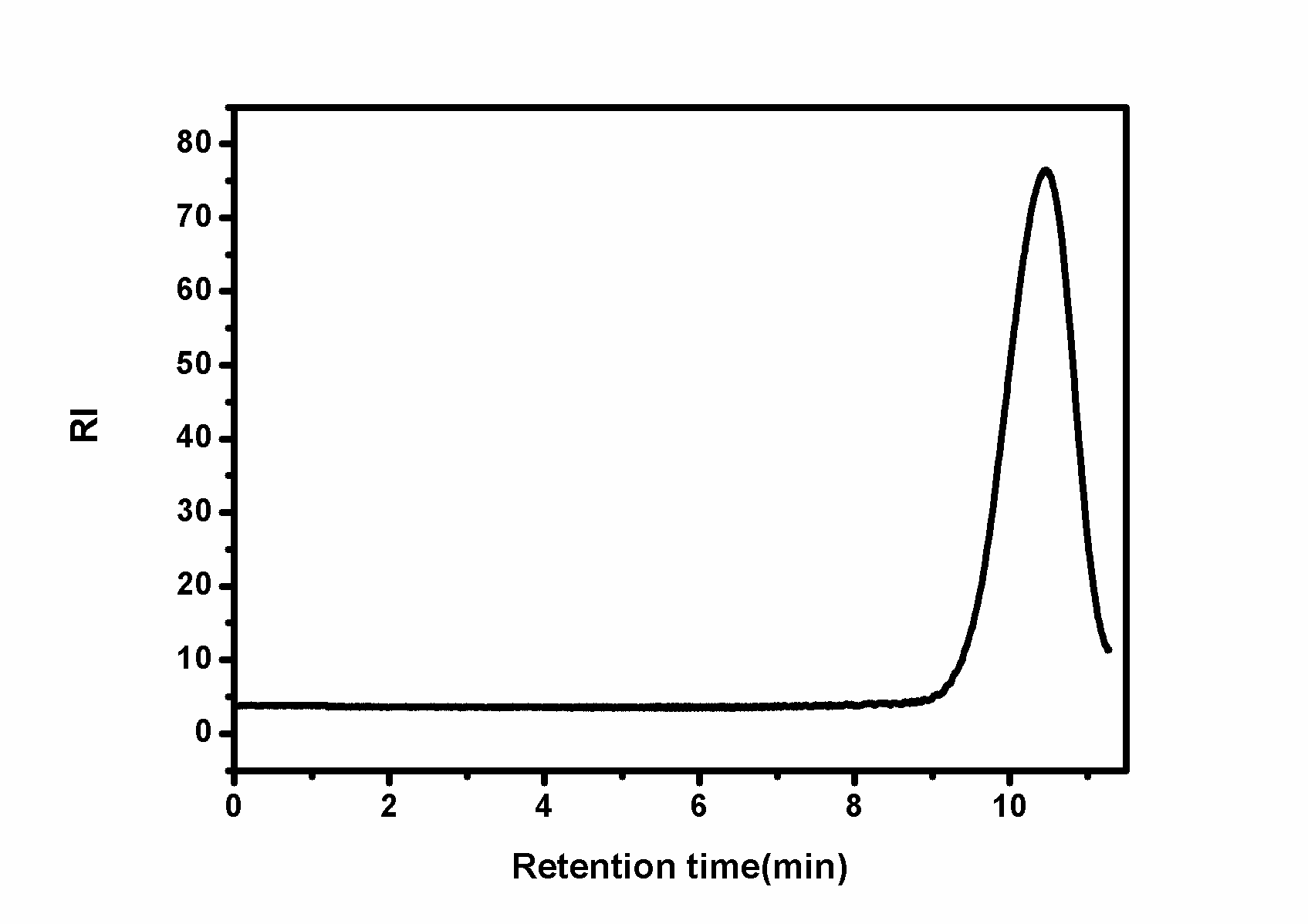


**Figure S7.** The ^1^H-NMR and GPC of PEO Macro-initiators


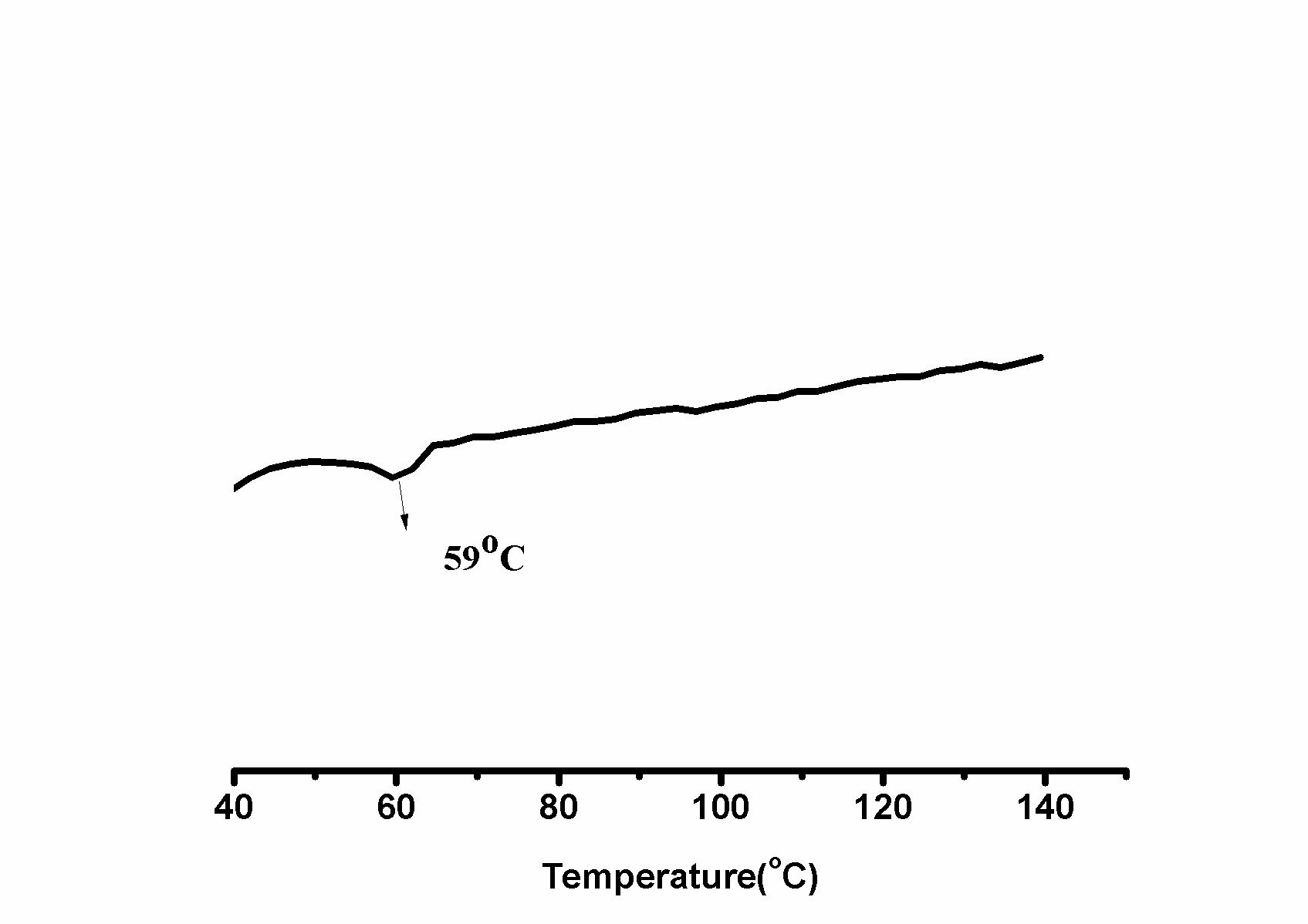


**Figure S8.** The DSC curve of un-crosslinked block copolymer PEO_11_-*b*-PMA(rChal)_12_
